# A conceptual ethical framework to preserve natural human presence in the use of AI systems in education

**DOI:** 10.3389/frai.2024.1377938

**Published:** 2025-01-21

**Authors:** Werner Alexander Isop

**Affiliations:** Independent Researcher, Graz, Austria

**Keywords:** ethical framework, education, artificial intelligence, trustworthy AI, ethical behavior, visual representation, human-centric

## Abstract

In recent years, there has been a remarkable increase of interest in the ethical use of AI systems in education. On one hand, the potential for such systems is undeniable. Used responsibly, they can meaningfully support and enhance the interactive process of teaching and learning. On the other hand, there is a risk that natural human presence may be gradually replaced by arbitrarily created AI systems, particularly due to their rapidly increasing yet partially unguided capabilities. State-of-the-art ethical frameworks suggest high-level principles, requirements, and guidelines, but lack detailed low-level models of concrete processes and according properties of the involved actors in education. In response, this article introduces a detailed Unified Modeling Language (UML)-based ancillary framework that includes a novel set of low-level properties. Whilst not incorporated in related work, particularly the ethical behavior and visual representation of the actors are intended to improve transparency and reduce the potential for misinterpretation and misuse of AIS. The framework primarily focuses on school education, resulting in a more restrictive model, however, reflects on potentials and challenges in terms of improving flexibility toward different educational levels. The article concludes with a discussion of key findings and implications of the presented framework, its limitations, and potential future research directions to sustainably preserve natural human presence in the use of AI systems in education.

## 1 Introduction

The broad use of AI systems (AIS) in daily life extends beyond the educational field and has been well-established for many years. In 2016, large companies such as Amazon, Apple, Deep Mind, Google, Facebook, IBM, and Microsoft initiated the “Partnership on Artificial Intelligence” to influence the trajectory of future technologies in our industrial society (Kejriwal, 2022). This collaboration has led to an increased presence of AI technology in commercially available products. Early AI-powered products included Amazon's Alexa, Apple's Siri, Google's Assistant, and Microsoft's Cortana, which served as speech-based natural interfaces (López et al., 2018) and were heralded as the “next generation of virtual personal assistants (Kepuska and Bohouta, 2018).” Since then, there have been significant advancements in computational power (Hwang, 2018), leading to a new era of AIS. Innovations have emerged not only from large companies, such as Microsoft's CoPilot, but also from collaborative ventures that initially offered their services for free, leading to the development of ChatGPT (Jungherr, 2023). More recent generative AI technologies, developed by OpenAI (Baidoo-Anu and Owusu Ansah, 2023; Kasneci et al., 2023; Sallam, 2023) and Hugging Face (Syal, 2020), have become widely accessible, profoundly affecting teaching and learning. These solutions predominantly include chatbots and similar applications that utilize Large Language Models (LLM) to foster the creation and advancement of “friendly AI” in education.

The use of AIS in education, in particular intelligent information systems or robots, has seen a significant increase in popularity over the past two decades (Zhai et al., 2021; Chen et al., 2022). While years ago, researchers predicted that AI would strongly affect the existential future of life (Barrat, 2013; Arney, 2016; Müller and Bostrom, 2016), a corresponding impact on education, with a shift toward human-centered and “human-friendly” AIS, was to be foreseen (Xieling et al., 2023). The range of applications has broadened from simpler software-based tools that assist with teaching administration, instruction, and learning (Chen et al., 2020), to advanced applications for adaptive learning, virtual classrooms, or intelligent tutoring robots (Huang et al., 2021), marking an increasingly widespread adoption of AIS.

With regards to learning strategies, the use of AI-powered assistive systems has also become increasingly popular. Recent discussions have highlighted benefits for various methods, such as blended, lifelong, or collaborative learning (Chen et al., 2022; Sanchez Ruiz et al., 2023; Mhlanga, 2023). However, recent work investigates challenges, valid concerns, and pitfalls (Qadir, 2023). Concurrently, Nguyen et al. (2023) have stressed concerns about insufficiently addressed ethical issues in education. The significant growth of applications across interdisciplinary fields underlines the vast capabilities of AIS. Nonetheless, the widespread implementation of such systems to support social educational processes also necessitates accompanying social and human-centric concepts that include responsible and ethical strategies (Kasneci et al., 2023).

During the COVID-19 pandemic, the critical importance of social presence and interactions among natural human actors in remote education, work, and life became evident (Dwivedi et al., 2020). More recent studies highlight a significant decline in educational quality due to the absence of physical interactions in online collaborative environments (Kalmar et al., 2022; Baber, 2022). When considering the use of AIS in such contexts, Alam (2021) posits that corresponding technologies could potentially replace didactic roles. However, they propose that AI will act more as a reformer and facilitator of educational use cases at the operational level. Tuomi (2020) emphasizes that for improving competitive digital skills and competencies in an AI-enabled future, the use of essentially AIS will be required. Nevertheless, it is suggested that “the learning outcomes do not depend on technology. It depends on how the teachers can use technology in pedagogically meaningful ways.” Furthermore, a great potential in compensating learning difficulties and supporting teachers is highlighted. The work of Fischer (2022) contrasts two basic ethical design approaches of “Humans for AI” and “AI for Humans” and questions which one to prefer in the presence of technology growth in a digital age. It is highlighted that potential negative effects should be avoided rather than treated as unfortunate but unavoidable side effects and conclude with an “AI for Humans” perspective. Accordingly, Pagano et al. (2023) project modern digital tools into the two contexts of artificial intelligence and intelligence augmentation and highlight future challenges in finding a balance to ensure that AIS are used responsibly, safely, and ethically. Furthermore, Ninaus and Sailer (2022) stress the importance of balancing human and AI-driven decisions, concluding that humans remain crucial “at many stages in the process of designing and using artificial intelligence for education.” These studies highlight potential negative impacts of AIS on society, both now and in the near future, if a social, human-centric, and sustainable strategy is denied (Fernández Aller et al., 2021). Consequently, since the use of AIS generally demands responsible perspectives (Kellmeyer et al., 2022), the goal of the presented framework is to facilitate responsible and ethical implementations in education (Halaweh, 2023).

**Figure 1:**
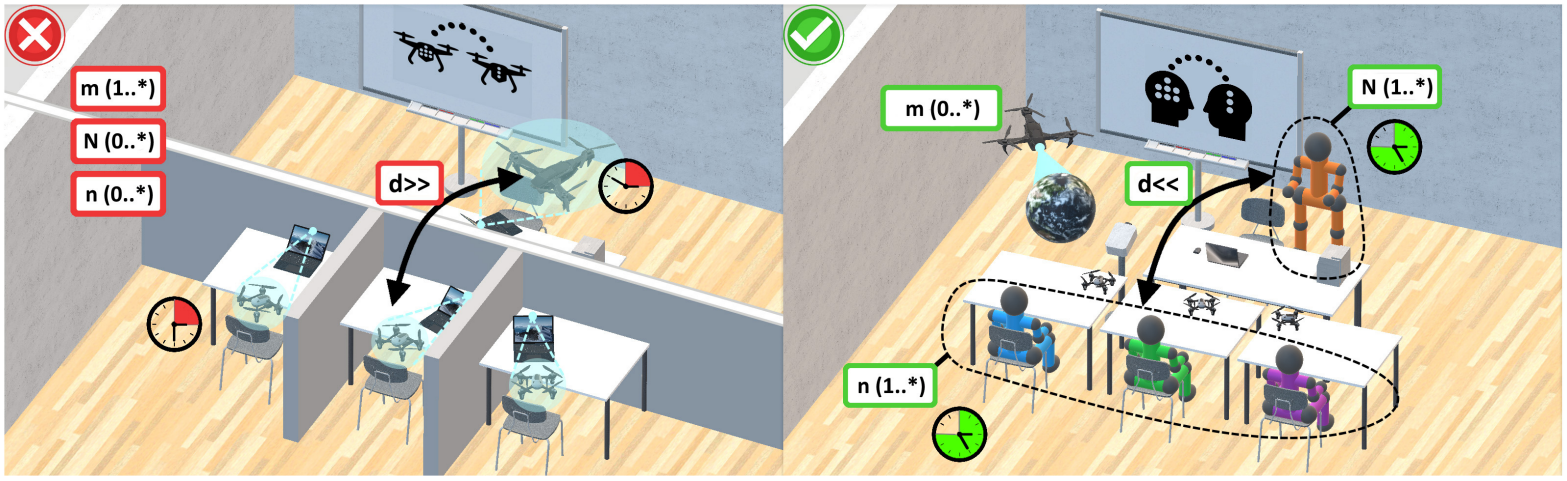
A conceptual ethical framework in the use of AI systems in education. On the left, an unethical use (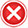) is depicted, with all real natural human actors being substituted by *m*(1..*) purely virtual AI systems in a predominantly asynchronous (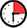) and distant (*d*>>) setting. On the right, a contrasting ethical use (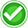) is depicted, preserving at least *N*(1..*) real natural human educators and *n*(1..*) learners, supported by *m*(0..*) AI systems, in an overwhelmingly synchronous (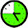) and co-located (*d* < < ) setting.

In the following section, state-of-the-art concepts and frameworks for the ethical use of AIS within education are analyzed. Addressing the deficiency of detailed processes and according properties of the involved actors, the concept of an ancillary ethical framework is introduced. It considers a specific interactive process of teaching and learning from which a novel set of ethical low-level properties is derived. Emphasizing the human-centric approach, a human senses taxonomy is extended to substantiate the development of the set of properties. The set describes ethically essential properties such as role, multiplicity, behavior, synchronicity, location, and visual representation of the involved actors. The importance of these properties is underscored by discussing unethical use that may occur if the properties are ignored in typical educational processes (Figure 1). Underlining the implementable character of the ethical framework, ultimately concrete practical guidelines for AIS in education are formulated. These serve as a foundation for discourse, with the overarching aim of minimizing the potential for misinterpretation and misuse.

## 2 Related ethical concepts and frameworks

Developing a complete and comprehensive framework that encompasses all ethical dimensions of utilizing AIS in education is time consuming and challenging, if not unfeasible. Bennett and Maruyama (2021) identifies two primary challenges associated with enforcing “ethical AI.” Firstly, ethical considerations often appear highly subjective within a specific field, context, or application, diverging from a broader consensus beyond a particular community. Secondly, AIS have operated inside well-defined boundaries, to simply search for systematic solutions without any ethical concerns, for many years (Fradkov, 2020). The application of AIS and machine learning across various fields has surged recently, with a vast increase in the availability of training data for AI-driven education (Munir et al., 2022). Moreover, along with the COVID-19 crisis, a substantial rise in distance learning (Adedoyin and Soykan, 2023) rapidly advanced the capabilities of AIS quite recently to the point that natural human actors have become “in a way, dispensable in some jobs (Flores-Vivar and Garćıa-Peñalvo, 2023).”

In response to the future development of AIS and its potential societal impacts (Tuomi, 2018), the European Union (EU) made efforts to establish “Ethics Guidelines for Trustworthy AI” in 2019 (Smuha, 2019). Committed to the “2030 Agenda” and its set of sustainable development goals (SDGs) of the United Nations, a key objective is to “promote an inclusive and sustainable AI strategy, rather than a strategy with a narrow focus on competitiveness (Fernández Aller et al., 2021).” The term “trustworthy AI” serves as an umbrella term under which an independent High-Level Expert Group (HLEG) was established by the European Commission (HLEG, 2019). The HLEG identified three fundamental ethical high-level principles: “lawful AI,” “ethical AI,” and “robust AI.” From these principles, requirements were developed to be assessed throughout the lifecycle of any AIS. In 2021, the European Commission announced a proposal for regulations on harmonized rules and liability rules for AI (European-Commission, 2021; Madiega, 2021), which recently entered a transitional period (Nikolinakos, 2023).

In recent years, the EU has made significant efforts, leading the HLEG to develop a robust and comprehensive framework for justifying the use of AIS in daily life. The proposed framework, which is high-level and adopts a risk-based classification, concentrates on system capabilities, outlining what systems could, should, and might do (European-Commission, 2021). Additionally, it includes an assessment list for operationalization, grounded in ethical high-level principles and key requirements. Moreover, state of the art regulations of the European Parliament contain indications on forbidden usages, like for example “emotion recognition systems.” They also define according high-risk usages to prevent “profiling” in the context of education (European-Parliament, 2024). However, other relevant use cases for mimicking or reproducing natural human behavior, like expression of emotions to increase the overall educational performance (Rodrigo-Ruiz, 2016; Stark and Hoey, 2021; Pusparini and Rahmajanti, 2023), are not explicitly addressed.

Consequently, an AI system deemed ethically sound in one process (He et al., 2023) could cause severe social harm in another (Milano et al., 2023). As it seems more important to understand why and for what AI technology is used, than how it is used (Tuomi, 2018), this high-level classification's shortfall, coupled with the absence of practical low-level properties, is a concern echoed in other works. Previous contributions to AI ethics have pointed out the absence of “professional history and norms,” “proven methods to translate principles into practice,” and “robust legal and professional accountability mechanisms,” questioning the utility of consensus on high-level principles (Mittelstadt, 2019). Related work admits a globally converging consensus about ethical principles, however, explicitly highlights a “substantive divergence in relation to how these principles are interpreted, why they are deemed important, what issue, domain or actors they pertain to, and how they should be implemented (Jobin et al., 2019).” Besides of emphasizing that ethical principles could only serve as a starting point, the work of Whittlestone et al. (2019) rises awareness of tensions regarding “ambiguities and knowledge gaps.” Further developing high-level principles to become more practical, Rothenberger et al. (2019) evaluated an early set of ethically relevant properties by their importance for industrial applications. Stressing ethical tensions between - and significant implications on - the roles of natural human teachers and robotic teachers in education, Newton and Newton (2019) proposed a code of practice. A more general ethical evaluation of AI guidelines was conducted later on, particularly stressing “a stronger focus on technological details of the various methods and technologies in the field of AI” as important requirement (Hagendorff, 2020). As later works investigated ethical frameworks for AI-driven educational technologies (Ashok et al., 2022), additional guidelines were drafted to support educators by the European Commission (EC-Directorate-General-for-Education-Youth-and-Culture, 2022). Such are supposed to put more emphasize on practical properties for teaching and learning. A more recent expressive summary of general state-of-the-art AI guidelines, ranging from high-level principles to requirements for responsible systems, is presented by D́ıaz-Rodŕıguez et al. (2023). Besides of placing emphasis on what each requirement for trustworthiness in AI stands for, still the relevance for the need of “regulatory directives that establish what, when and how AIS can be adopted in practical applications” is highlighted. More recent related work conceptualizes and establishes a set of ethical principles to inform and guide stakeholders, particularly in education (Nguyen et al., 2023). However, these concepts and frameworks remain predominantly high-level, lack concrete practical guidelines and low-level models for implementation, or do not incorporate essential low-level properties for education (Li et al., 2023).

To date, to the best of the authors' knowledge, an ethical framework with detailed processes and according low-level properties, particularly incorporating the behavior and essential visual properties of the involved actors, is missing. In this context, also proven methods for implementation and direct guidelines are still absent in education. In response, this article conceptualizes an ancillary framework for the ethical use of AIS in education. Utilizing UML, the starting point is a specific intended process of teaching and learning, modeled down to detailed use cases. A novel set of ethical low-level properties is derived, revealing potential for misinterpretation and misuse. Moreover, ethical guidelines, and comprehensive workflows are provided to practically justify the use of AIS for an intended process in education.

## 3 Ancillary conceptual ethical framework

The “Requirements of Trustworthy AI,” outlined by the European Commission, serve as a vital foundation for the ancillary framework presented. The ethical requirements include: Human agency and oversight (HAO), Technical robustness and safety (TRS), Privacy and data governance (PDG), Transparency (TRA), Diversity, non-discrimination, and fairness (DNF), Societal and environmental wellbeing (SEW), and Accountability (ACC). The HLEG provides a high-level description of these requirements and related aspects (HLEG, 2021). In contrast, this article extends the high-level risk-based assessment of AIS and related aspects by presenting the design of a ancillary framework, including a set of low-level properties. It incorporates ACC and TRA as some of the most essential principles (Rothenberger et al., 2019; D́ıaz-Rodŕıguez et al., 2023; Yu and Yu, 2023), conceptually analyzing and extending existing high-level regulations and requirements (European-Commission, 2023; HLEG, 2021). With a focus on HAO, EU guidelines for teaching and learning (EC-Directorate-General-for-Education-Youth-and-Culture, 2022) are contextualized within “lessons” or “lectures” that relate to the interactive process of teaching and learning to achieve one essential goal of developing skills and competencies (Zamora and Zamora, 2022). The process is UML-based and includes typical exemplary use cases in education, also found in earlier virtual classrooms (Adewale et al., 2012), but also more recent online teaching and learning management systems (Adedoyin et al., 2023). Consequently, a novel set of ethically relevant properties is derived, emphasizing the behavior and particularly an ethical visual representation of all involved actors. Overall, actors in an intended process are then characterized by their role, multiplicity, behavior, location, synchronicity, and visual representation. To underscore the practical character of the framework and the significance of its set of low-level properties, the article highlights unethical uses that may arise if these identified properties are disregarded. To prevent potential misuse and minimize misinterpretation, ethical guidelines and a comprehensive workflow, based on the extended EU requirements, are proposed. Thus, the essential goal of the ethical framework is to foster discussion by defining more detailed processes, low-level properties of the involved actors, and supporting practical guidelines, to preserve natural human presence in the use of AIS in education.

### 3.1 Guidelines for teaching and learning

In addition to state-of-the-art high-level requirements, the presented framework is based on guidelines that provide more details for questioning the use of AI in education (EC-Directorate-General-for-Education-Youth-and-Culture, 2022). The European Commission's work addresses relevant ethical aspects of AI use, with a particular focus on teaching and learning. It includes future skills that educators need to assess the ethically justifiable use of AIS. Furthermore, it provides examples to aid in the assessment of ethical aspects of using such systems. The guidelines also include a glossary to explain complex technical or scientific terms. On one hand, the extensive effort of the related work must be recognized and acknowledged for its value in conceptualizing this article. On the other hand, the examples seem to guide educators on questioning EU requirements when AIS are used in less detailed scenarios. Besides, concrete recommendations or supporting workflows to facilitate the assessment of whether the use is justified are missing. Especially from an interdisciplinary perspective, with potential knowledge gaps and a lack of detailed descriptions of ethically important low-level properties, it may become “difficult to pin down all possible ethical implications (Köbis and Mehner, 2021).” Therefore, this article suggests that the current guidelines place too much responsibility for assessing the ethical use of AIS on educators.

As a first step, this article investigates room for improvement by detailing a typical interactive process in education. Subsequently, it reveals the necessity of defining more details about all involved actors and related missing low-level properties. This ultimately results in practical guidelines and workflows. In addition to providing a more detailed AIS-supported interactive educational process, the proposed framework adopts a UML-based abstraction related to a specific field, with characteristic specific goals and the underlying use cases of a specific intended process. For instance, “teaching” is a broad term and may be interpreted as a common everyday use case involving AIS beyond the field of education (Lin and Lin, 2014). Moreover, “teaching” has a multifaceted and potentially ambiguous meaning inside the field of education. Ranging from earlier interactive teaching-studying-learning perspectives (Kansanen, 1999), to more recent and simple interpretations of facilitating knowledge transfer between students in higher education (Peng et al., 2021), up to developing (digital) skills and competencies (EC-Directorate-General-for-Education-Youth-and-Culture, 2022), it may be interpreted in various forms. Similarly, the term “supporting students” is too unspecific since it may hold a variety of individual underlying goals. Consequently, the proposed interactive educational process is grounded in a more nuanced definition of the field and also encompasses typically required low-level use cases and a finer characterization of the involved actors. The importance of these details is asserted here and will be further elaborated upon in the remainder of this article. At this juncture, a general summary of the high-level identified issues and the corresponding requirements to the conceptual framework is provided as follows:

- A detailed definition of the concrete field and underlying concrete goals can serve as an essential starting point for a more clear description of intended educational processes at a lower level.
- A detailed definition of the interactive process of teaching and learning, including a dedicated system, actors, roles, and use cases, can significantly enhance clarity by providing a more concrete description.
- A detailed characterization of actors is crucial, with a fundamental distinction between natural human actors and AIS being a significant aspect within an educational process. However, conceptualizing unethical examples for using AIS has revealed additional low-level properties that could greatly clarify typical processes in education. These properties pertain to the behavior and specifically the visual representation of all involved actors.
- Concrete workflows and recommendations: Current regulations offer a solid foundation for considering which ethical aspects are even relevant at higher level. However, providing direct recommendations and additional workflows could further enhance practicability.

### 3.2 Deriving the set of properties

To more closely inspect the identified issues, the next step introduces a detailed UML-based educational process. Starting with a specific field at a higher level, the concept is developed top-down to a lower level, encompassing system boundaries, essential overall goals, and typical use cases in education.

#### 3.2.1 Goals, systems, and actors

Artelt and Kunter (2019) provide a comprehensive summary of typical use cases and according sub goals involved in teaching and learning. They address the design of lessons or lectures, focusing on the “delivery of teaching content (Peng et al., 2021)” detail this as the cognitive processes of “providing knowledge” and “receiving knowledge,” which (Tsankova and Manolova, 2022) contextualize within education.

A broader perspective on related essential use cases and connected goals as part of an AIS-supported “interactive process of teaching and learning” is given by Munna and Kalam (2021). A very basic and early definition of “learning” emphasizes that “Many, but not all, forms of learning have to do with acquiring knowledge—either knowledge that something is the case or knowledge how to do something (White, 1998).” A more recent understanding of related “competencies” describes them as “knowledge, skills, attitudes, values, motivations, and beliefs people need in order to be successful in a job (Selvi, 2010).” Additionally, Musial et al. (2012) connect the terms “teaching” and “skills” with higher granularity as the “practice implemented by a teacher aimed at transmitting skills (knowledge, know-how, and interpersonal skills) to a learner, a student, or any other audience in the context of an educational institution.” Moreover, they state that “teaching is closely related to learning, the student's activity of appropriating this knowledge.” Also, Bialik et al. (2015) further reflect on skills and competencies, whereas they suggest that “skills relate to the use of knowledge and engage in a feedback loop with knowledge,” whereas “competency, on the other hand, is defined as the set of knowledge, skills, and experience necessary for future, which manifests in activities.” Complementary, Zamora and Zamora (2022) mention that “for some, skills are a combination of the knowledge, abilities and experience they have obtained both before entering the profession and during their employment.” Also, they reflect on an interplay between teaching and “facilitation of learning,” while in this context a recent work of Rone et al. (2023) strictly emphasize the importance of engaging motivation and classroom participation of learners. In education the interactive process of teaching and learning, particularly with the support of AIS, lead to a significant paradigm shift in recent years (Gentile et al., 2023). A more modern understanding of interactive teaching processes and required skills and competencies is introduced by Albrahim (2020); Blane (2021); Garcia (2024) and Ng et al. (2023b). Accordingly, still vital learning activities (Jakavonytė-Staškuvienė and Mereckaitė-Kušleikė, 2023; Gericke et al., 2023), but also future (digital) learning processes and their required skills and competencies are discussed by Abendan et al. (2023). Thus, important future-proof goals, as part of interactive blended teaching and learning, particularly involve the development of skills and competencies in the digital age (Marr, 2022; Thornhill-Miller et al., 2023) also including Extended Reality and Metaverse applications (Jagatheesaperumal et al., 2024; Pregowska et al., 2024).

**Figure 2:**
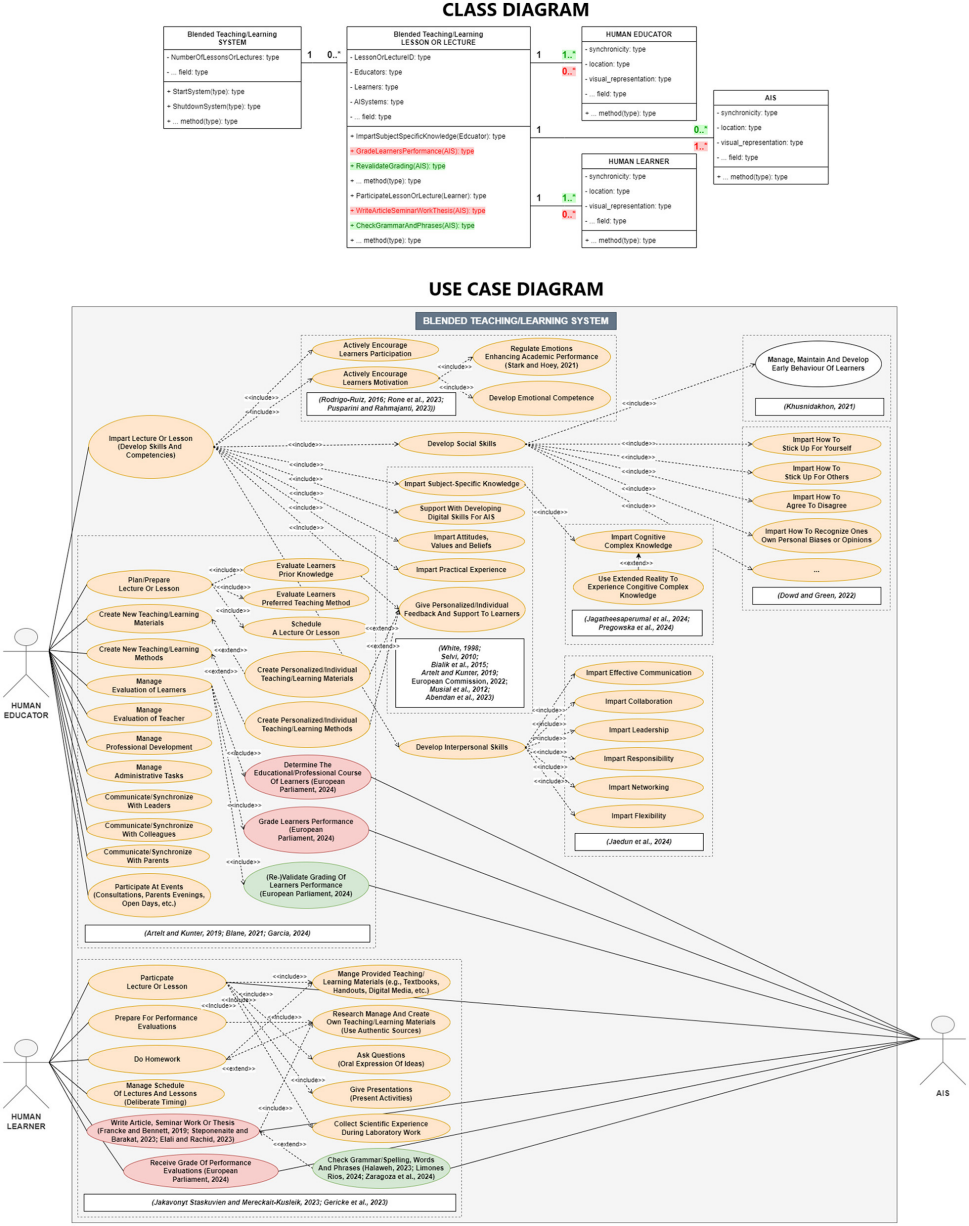
UML model of an AIS-supported blended teaching/learning system as a typical system in education. One essential goal is to develop skills and competencies during the interactive process of teaching and learning. At the top, a class diagram depicts the involved actors/roles and exemplary ethical or unethical behavior (methods). At the bottom, a use case diagram is represented, including exemplary ethical use cases of AIS depicted in green and unethical ones depicted in red.

Consequently, with the aim of offering more precise and practical definitions, “Education” is selected as a specific field, encompassing one essential goal of “developing skills and competencies.” In this field, the most apparent process to achieve the development of skills and competencies is defined as “the interactive process of teaching and learning,” including human actors, a blended teaching/learning system (Bhadri and Patil, 2022; Janse van Rensburg and Oguttu, 2022; Venkateswari, 2024), AIS and a wide array of corresponding use cases (Figure 2). Emphasizing an UML-based approach, any actor is in a specific role, whereas remarkably any use case can be accomplished by exhibiting certain behavior. Ethical behavior is also a well-known concept in digital AI-driven contexts, since “electronic interactions encapsulate both human-machine and machine-machine interactions (Buytendijk, 2014; Ashok et al., 2022).” These studies emphasize the importance of transparent and ethical behavior in processes where natural humans interact with machines. Therefore, ethical behavior is considered an essential additional low-level property of the proposed conceptual framework.

In summary, the proposed framework aligns with the previously discussed top-down definitions. Closely connected to the UML, it identifies “Education” as the field, “development of skills and competencies” as one essential overarching goal, and the AIS-supported “interactive process of teaching and learning (lessons or lectures)” with its various use cases. Furthermore, it involves human educators, human learners, and AIS as actors in specific roles which must exhibit ethical behavior while accomplishing designated use cases. Figure 2 gives an overview on most essential use cases for typical interactions of human educators, learners, and AIS with a blended teaching/learning system in school education. The objective is to support a systematic and model-based concept, refined to a granular level, that is implementable. Thus, ancillary to a risk-based assessment of any AIS at a higher level, this framework may facilitate the concrete ethical justification of related low-level use cases.

#### 3.2.2 Role and multiplicity of actors

In the previous section, essential preliminary definitions of the conceptual framework were identified to frame a concrete process. However, the framework still lacks detailed characterization of the involved actors. In response, the low-level human-sensing taxonomy (HST), introduced by Teixeira et al. (2010), is incorporated to facilitate an ethical use of AIS in the presence of natural human actors. In the context of typical educational processes, it is extended to a more detailed set of low-level properties. With the types of actors in their according roles as a starting point, it is possible to even distinguish between natural human educators, learners, and AIS. This distinction is crucial, as future AIS may be fully capable of behaving like natural humans, potentially leading to unethical mimicking or replacement. Whilst supporting or augmenting is a role widely accepted by the educational community, it is important that AIS do not replace natural human actors (Flores-Vivar and Garćıa-Peñalvo, 2023). Consequently, the first UML-related key property emerges, describing different “roles of actors,” also depicted in Figure 2. Further, in alignment with the HST, the set adopts the “number of people in an environment (count)” as the UML-based “mulitplicty” (Figures 1, 2). Hence, as an intermediate result, the framework defines three different roles of actors with the according multiplicity:

- N (1..*) human educators (natural human actors, e.g., professors, lecturers, teachers, trainers, instructors, and similar), leading a lesson or lecture, as part of the interactive process of teaching and learning, to develop competencies and skills. Also supporting the learners, they are interactively leading and conducting the goal of developing skills and competencies by providing knowledge. Thus, they predominantly act in a leading role.
- n (1..*) human learners (natural human actors, e.g., students, pupils, trainees, apprentices, and similar), that are participating, and contributing to, the interactive process of teaching and learning.
- m (0..*) AIS (artificially intelligent actors, e.g., robots, bots, pre-trained transformers, recommender systems, and similar), supporting the natural human educators and learners with the goal of developing skills and competencies. Thus, they may predominantly act in a supporting role.

Natural human educators, human learners, and AIS, as the principal actors, may interact with a dedicated teaching/learning system in their specific roles, contributing to the overarching goal of developing skills and competencies. The interactions of the AIS are modeled with either ethical or unethical use cases (Figure 2). Combined with a well-defined role and multiplicity, the sum of these properties are intended to significantly clarify a potential process in education already. However, at the same time they question whether the actors are interacting simultaneously within a particular system, or if a potential for misuse must be considered. Concerns may be negligible in traditional lessons or lectures, where actors are physically close, and their presence and role can be readily identified. Nevertheless, issues may surface in typical distance learning settings, where actors are interacting online, over wider distances. To address related issues, it is proposed that essential properties of an actor's presence be identified to foster ethical justification.

#### 3.2.3 From human presence to the visual representation of actors

The full spectrum of natural human presence is a multifaceted, complex, and long investigated topic (Segal, 1996), certainly exceeding the boundaries of this article. Nevertheless, essentially the multi-sensory perception of human presence can be described by the process through which humans interpret sensory information to construct their individual experiences of the real world. Furthermore, in addition to the HST, Cornelio et al. (2021) present an extensive review of recent technological advances related to the main human senses: vision, audition, touch, olfaction, and gustation, whereas research states that vision is the “most valued” (Enoch et al., 2019) and “most important” (Hutmacher, 2019) sense, being the sense humans mostly rely on. Consequently, the most relevant spatial and temporal, along with the visually recognized strongest, properties of any involved actor, are synthesized in the remainder of this section, leading to the “visual representation.”

Reflecting on essential temporal and spatial properties in an educational context, the concept of blended teaching/learning provides widely accepted definitions. It distinguishes clearly between synchronous (online) and asynchronous (offline) educational settings (Cleveland-Innes and Wilton, 2018) leading to the property of “synchronicity.” In synchronous settings, educators and learners are virtually present simultaneously, whereas in asynchronous settings, they are not. Moreover, blended teaching/learning distinguishes between co-located (face-to-face) and distant (distance learning) settings representing the property of “location.” In a co-located setting, educators and learners are physically present, contributing to the interactive process of teaching and learning at close distances (*d* < < ). This may pertain to a typical school lesson in a classroom or a traditional lecture in a lecture hall. Conversely, in a distant setting, educators are typically connected over greater distances (*d*>>) through distance learning and, unlike face-to-face settings, are not present in the same physical space (Ayu, 2020).

**Figure 3:**
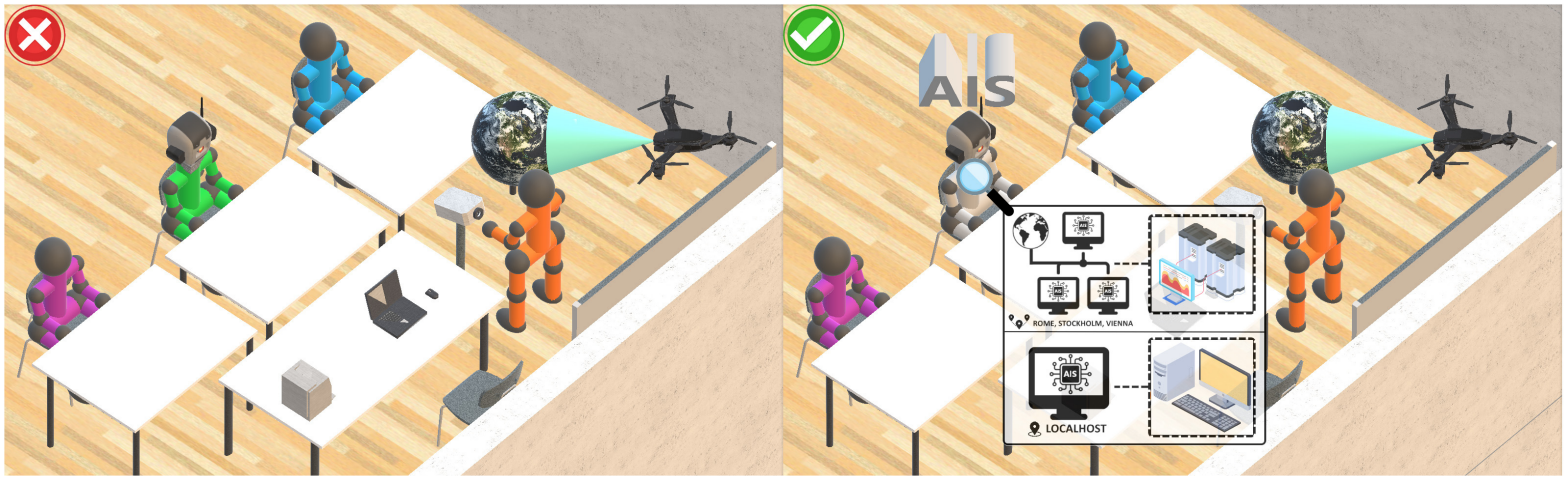
Exemplary ethical and unethical use cases, addressing the role property. On the **(left)**, an AIS is not obviously identifiable as such, revealing potential for misinterpretation and misuse. On the **(right)**, the AIS is explicitly labeled and identifiable as such. A conceptual visual aid indicates details about the visual representation of the AIS (distributed/omnipresent or local) on demand, improving TRA.^1^

In addition, the “embodiment” of the involved human actors and AIS may help to better define physical presence (Wainer et al., 2006; Mollahosseini et al., 2018), or, at least, its visual perception for distant settings (Bonfert et al., 2021). In contrast, any missing representation of embodiment, may lead to omnipresence, also causing issues with trustworthy AIS in similar settings (Kim et al., 2018; Sathikh et al., 2022). In the same way it may reduce transparency and trust in educational processes. Moreover, if the representation of embodiment is entirely missing, compared to any obviously (visually) represented actor, it may become significantly harder to distinguish between the roles of actors (HAO, ACC). Essentially, any required visual indication of the actual physical embodiment of the AIS could help to explain a more concrete spatially limited reference, better clarifying the boundaries of the AIS to improve TRA (Figure 3).

**Figure 4:**
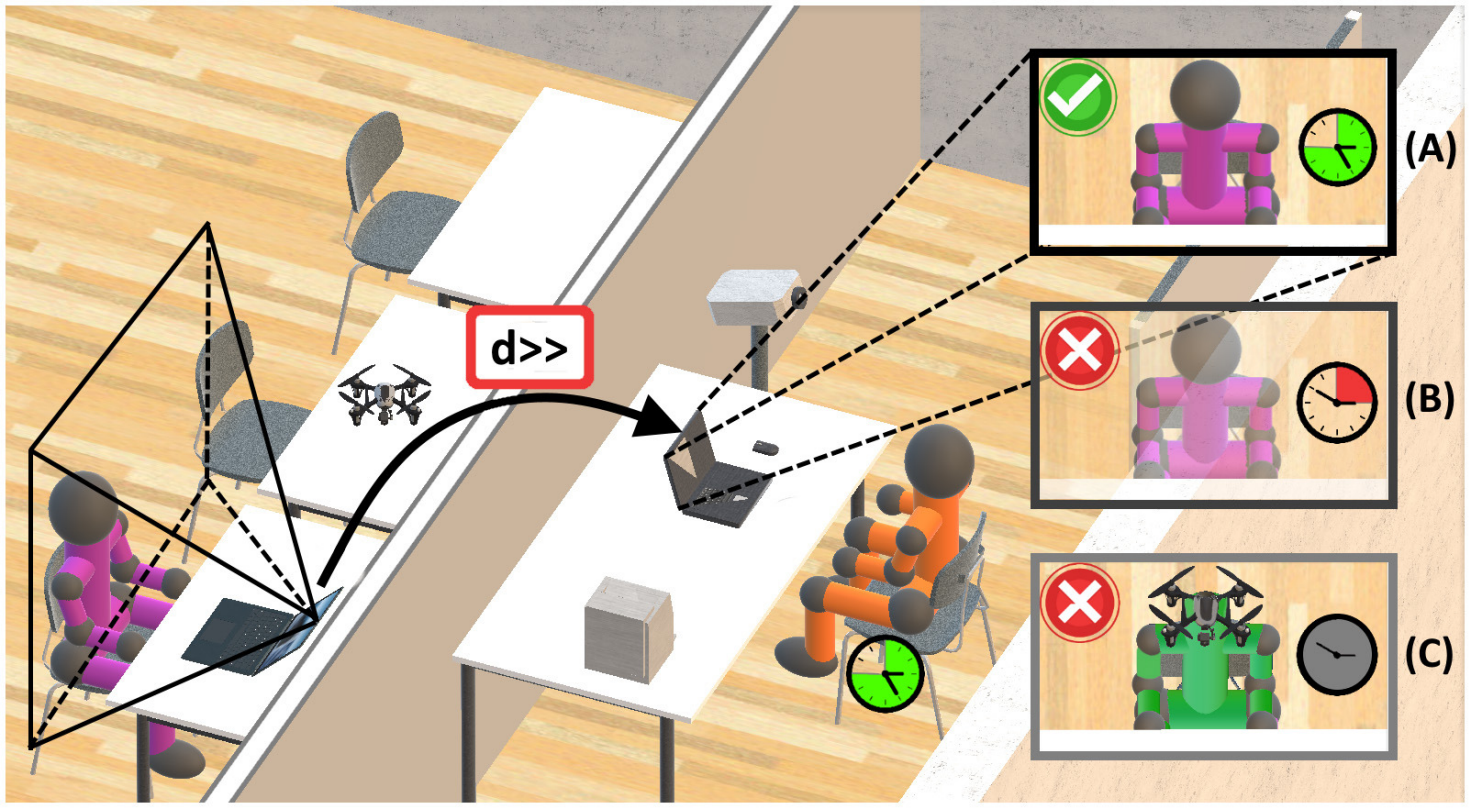
Requirements on an actor's “virtualization” as ethical visual representation to facilitate HAO, TRA, and ACC. A real natural human actor (orange), is perceiving the visual representation of a second actor (purple) during distance learning. In the ethical use case **(A)** synchronicity, true relative scale, and accurate colors of the embodiment are fulfilled, whereas case **(B)** lacks synchronicity and case **(C)** does not preserve coherence of embodiment.

Under the assumption that any natural human actor's visual sense is the most important (Hutmacher, 2019), the resulting definition of a visual representation, combining the three properties synchronicity, location, and embodiment, requires more specific definitions from the field of visualization. Being particularly important for distance learning, the goal is to characterize if the visual representation of the involved actors is real, virtual or something in between, posing a strong indicator for their presence. A rather interdisciplinary perspective on physical real and visual virtual presence is given by Lee (2004), whereas a long established taxonomy emphasizes visual properties of an actor, ranging from real to virtual (Milgram et al., 1995a). An according widely accepted scale is the RV-continuum (Milgram et al., 1995b), “revisited” more recently by Skarbez et al. (2021). More recent work focuses on the definition of design spaces that better clarify what is real and what is virtual in settings that are similar to the presented framework, however, they do not focus on detailed ethical considerations in the context of education, but investigate on the social relevance of a virtual assistants embodiment in everyday life (Kim et al., 2018). Moreover, whilst Lee et al. (2023) present design patterns for situated augmentation of physical referents, the work of Suzuki et al. (2022) puts a stronger focus on augmenting interactions between natural humans and robotic AIS. Additionally, an extensive review of definitions of a digital twin, describing similar transitions between physical and virtual entities, is provided by Semeraro et al. (2021). Still, the unambiguous definition of a property with focus on ethical use in education seems to be missing. Specifically important for distant settings, the visual representation of any actor may be characterized by three distinct cases:

- **Real representation**: The first case poses the real visual representation of any actor, whereas the embodiment is fully spatially coherent in a synchronous setting.
- **Virtualization**: The second kind of representation is between being either real or virtual. It involves any real embodied actor, being visually displayed (Milgram et al., 1995b) and requiring, above all, synchronicity. Second, at the same level of abstraction, the visualized embodiment must be given with true relative scale and accurate color. Since the actor is virtually represented, but must meet a strong relation to the real world at the same time, this representation is defined as the actor's virtualization. The required properties of a virtualization are depicted in Table 1 and Figure 4.
- **Purely virtual representation**: The third kind of representation, specifically important for an ethical perspective, lies on the opposite side of the RV continuum (Milgram et al., 1995b). It is purely virtual, since it is non-existing as such in the real world. The actor is either represented asynchronously (Table 1, “Purely Virtual—Case 1” and Figure 4B), or, if in a synchronous setting, the visualized embodiment (true relative scale and accurate colors) significantly diverges from the real world representation (Table 1, “Purely Virtual—Case 2” and Figure 4C).

**Table 1:** Coherence of synchronicity, location, and embodiment with the real world, to ethically classify an actors visual representation.

**Visual representation**	**Coherence of the related properties with the real world**		
	**Synchronicity**	**Location**	**Embodiment**
Real	✓	✓	✓
Virtualization	✓	❍	true rel. scale/accurate color
Purely virtual—case 1	✗	❍	❍
Purely virtual—case 2	✓	❍	✗

Symbols are indicating (✓) as “Coherent,” (✗) as “Non-coherent,” or (❍) as “Not relevant.”

Remarkably, a classification as virtualization requires, above all, synchronicity and at least a visually true relative scale and accurate color of the embodiment (Table 1). The location is noted to more clearly define a real actor and distinguish between co-located and distant settings, however not relevant for a virtualization since it is typically displayed with wrong absolute scale and non-coherent location during distance learning. The main purpose of the virtualization is to preserve the presence of natural human actors, particularly regarding the visual properties. If not considered, any actor may not be identifiable as such, in turn leading to a great potential for misuse. As a consequence, negative effects are to be foreseen regarding TRA, ACC, and HAO. Summarizing, the conceptual framework suggests the following essential low-level properties to classify a visual representation: The temporal coherence or synchronicity, reflecting the time difference between actors; the spatial coherence or location, which is the distance between actors; and a strong coherence of the embodiment, indicating if the actor even exists as such in the real world. Posing a substantial property for distance learning, the visual representation of an actor could help to better distinguish between ethical and unethical use.

### 3.3 Potentials for ethical use and unethical use and resulting guidelines

In the context of preserving natural human presence, the use of AIS is of course not entirely disadvantageous or poses harms to human interaction in general. Quite the opposite seems to be the case, if potential impacts on teaching and learning experiences are considered (Toksha et al., 2022; Kimondo et al., 2023). State-of-the-art AIS are able to enhance the acquisition of information by students or facilitate personal learning experiences, like support for inside classroom learning activities [e.g., “imparting complex cognitive knowledge” (Jagatheesaperumal et al., 2024; Pregowska et al., 2024)], enhancement of independent learning [e.g., “checking grammar, spelling, words, and phrases” Halaweh, 2023; Limones Ŕıos, 2024; Zaragoza et al., 2024], and mentoring and tutoring. Moreover, benefits of teaching AI itself across different educational levels is an essential future aspect in education (Long and Magerko, 2020; Kong et al., 2021; EC-Directorate-General-for-Education-Youth-and-Culture, 2022). As “Students are not merely consumers of AI applications, but creators of intelligent solutions which require teaching the AI concepts behind” (Ng et al., 2023a), related AIS are able to enhance non-technical students to generate machine learning models without computer science prerequisites. Thus, difficulties with the interactive process of teaching and learning AI, to develop future skills in the digital age, may be significantly reduced (Gresse von Wangenheim et al., 2021). Finally, Onesi-Ozigagun et al. (2024) provide a comprehensive overview on the enhancement of teaching and learning in the use of AIS. They suggest that AIS are able to enhance personal learning experiences [e.g., “creation of personalized/individual learning materials” (Zhou et al., 2020; Tiwari, 2023; Pesovski et al., 2024)], foster teaching and learning strategies [e.g., “creation of personalized/individual learning methods” (Kshirsagar et al., 2022; Tapalova and Zhiyenbayeva, 2022; Alam, 2023)], help to reshape assessment methodologies [e.g., “re-validation of grades” (European-Parliament, 2024)], and optimize administrative tasks (Tapalova and Zhiyenbayeva, 2022).

Besides of the tremendous potential of AIS to maintain, enhance or revolutionize education, many related works also highlight concerns and reflect on potentials for unethical (future) use of AIS. The remainder of this section contrasts the great positive potential of AIS with negative examples and underscores a responsible use. Likewise, the importance of the ethical framework's low-level properties is substantiated, and, moreover, their significance for the overall interactive process of teaching and learning. Building on the premise that a concrete detailed process can serve as a starting point, Section 2 motivated the need to differentiate between cases for ethical use. While ethical concerns about the use of AIS may not be justified for one process, they could be relevant for another. Therefore, modeling detailed processes and corresponding use cases can facilitate ethical decision-making. Referring to the educational process modeled in Figure 2, the significance of the previously considered properties (Section 3.2) is examined. Intentionally, first the potential for misinterpretation and misuse is discussed and illustrated if these properties are not considered. This is followed by the formulation of ethical guidelines, also connecting the properties to the high-level requirements of the EU regulations (HAO, TRA, DNF, and ACC), in response.

#### 3.3.1 Role

The role property, previously discussed in Section 3.2.2, is essential to even differ between human actors and AIS, thus maintaining HAO and TRA and preventing misuse (ACC) (Figure 3). Relevant use cases, beyond the field of education, include for example medical applications. Fiske et al. (2019) and Shuaib et al. (2020) express their concerns about robots replacing therapists or doctors, whereas more recent works address potential unethical roles of AIS in concrete medical educational settings (Cornwall et al., 2024). With focus on education, various works investigate on the replacement of educators with AIS in academia (Karki and Karki, 2023) or higher education, also being “better equipped to deliver assessments” and grading (Chan and Tsi, 2024). Also, Okulich-Kazarin et al. (2023) provide insights into the possibility of replacing teachers with AIS. Besides, once an AIS is implemented for a specific use case, concerns about a robust traceability, explainability, and avoidance of unfair bias of decisions imply potential unethical use. Strategies to counteract are suggested by HLEG (2021), whereas “algorithms used, should be documented to the best possible standard and decisions made by an AI system should be understandable” (TRA), while it is recommended to use “oversight processes to determine AIS' purpose, constraints, requirements and decisions in a clear and transparent manner” (DNF). However, these requirements and mechanisms also require concrete and detailed models to effectively prevent potentials for unethical use. As a strong indicator of potentials for unethical replacing roles and use cases is a gradual social deterioration (also highlighted by the works of Friedman, 2023; Frambaugh-Kritzer and Petroelje Stolle, 2024 and Indurkhya and Sienkiewicz, 2024), this article stresses that for many use cases in education, AIS can enhance learning experiences, however, “cannot replace the vital role of human interaction in education” (Zaman, 2024).

#### 3.3.2 Multiplicity

The basic considerations of Section 3.2.2 on the multiplicity are entirely adopted to determine if at least N (1..*) one natural human educator and n (1..*) one natural human learner are participating in the interactive process of teaching and learning. In this context, potentials for unethical use may include the full or partly replacement of natural humans by AIS (Fitria, 2023; Etiubon and Etiubon, 2023). Thus, determining the multiplicity of AIS with m (0..*), helps to distinguish if such are even involved into the overall process, including all ethical implications, or not. Supplementary, the multiplicity is modeled in Figures 1, 2.

#### 3.3.3 Behavior

**Figure 5:**
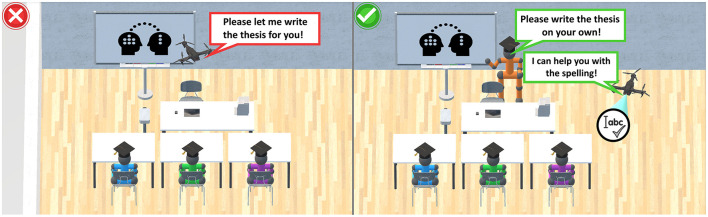
Exemplary ethical and unethical use cases, addressing the behavior of actors in their according roles. On the **(left)**, the AIS encourages unethical behavior in a replacing role (Francke and Bennett, 2019; Steponenaite and Barakat, 2023; Elali and Rachid, 2023). On the **(right)**, the AIS maintains responsible and ethical behavior in a supporting role, respecting “academic honesty (Halaweh, 2023; Limones Ŕıos, 2024; Zaragoza et al., 2024).”

Once the roles and the multiplicity are well defined, an ethically justifiable, exemplary behavior of all involved actors to accomplish use cases and overarching goals in the intended interactive process of teaching and learning is essential. Closely connected to the goal of developing skills and competencies it is a fundamental concept in education (Gunderman, 2002; Lumpkin, 2008; Khusnidakhon, 2021; Dowd and Green, 2022; Jaedun et al., 2024). Educators do not simply deliver teaching content without any self-reflection. Rather, they are challenged to show exemplary behavior to the learners as a role-model, also motivating and engaging active participation (Nasir and Hossain, 2023). If the behavior of any AIS contradict this concept, this may result in a significant negative effect, in the same way affecting HAO and SEW. A complementary example of misuse, emphasizing the interplay between role and behavior, is shown in Figure 5.

#### 3.3.4 Location

Although, co-located in presence participation of human actors in teaching and learning in-class can have significant positive impact on the educational experience and performance, a variety of modern educational processes involve distance learning. However, many facets regarding natural human presence are still hard to maintain over wider distances, from a social and interpersonal perspective (Leo et al., 2021; Anastasakis et al., 2023; Kornfield et al., 2021), but also from a technical-scientific perspective (Tsankova and Manolova, 2022; Dustova, 2023). Once improved, such facets could pose a vital basis for facilitating the overall quality of developing skills and competencies during distance learning. Nevertheless, in such settings, natural human presence may be more elusive and more prone to misuse (Figure 4), particularly with focus on HAO, TRA, and ACC. In these regards related work underlines “concerns with accountability, agency, and surveillance in online learning” (Seo et al., 2021), or stresses “a real danger of AI becoming pervasive in every sense where those involved may be exposed to risks without being aware of them” (Kamalov et al., 2023). Other works highlight issues with HAO and ACC, since (online) virtual AIS “will enable conversations that are hardly distinguishable from real conversations with humans, but they will also raise concerns about bias, transparency, and accountability (Piñeiro-Mart́ın et al., 2023).” Concluding, on one hand, the location of the human actors clearly effects the overall teaching and learning experience. On the other hand it can have great impact on the potentials for misuse of AIS in distant educational settings.

#### 3.3.5 Synchronicity

There is ongoing debate as to whether synchronous settings clearly provide benefits to the overall teaching and learning experience of students. If social aspects are concerned, related research investigates on the development of social networks and emotional interaction as part of an online monitoring framework for teaching and learning (Spadavecchia and Giovannella, 2010). Throughout analyzing text-message based interactions they found that dense social interaction can be also achieved in asynchronous online-settings, however socialization takes more time. Besides, Giovannella et al. (2011) found that teacher/learner interactions, like grading, can be seamlessly integrated, either in blended or pure online settings. Other related work suggests that both synchronous and asynchronous settings can enhance the overall quality of teaching and learning, whereas the preference which setting to choose strongly depends on the type of learner (Higley, 2013). Nevertheless, with focus on social aspects, related work concedes that during blended teaching/learning in general students “deem very important the collaboration/interaction among peers and with the teachers” (Giovannella, 2021) or, with synchronous settings, “feel a stronger sense of connection to their peers and instructor and stay engaged with course activities (Yamagata-Lynch, 2014).” Becoming specifically relevant during the COVID-19 pandemic, various works investigated on the efficacy of blended and hybrid learning, studying and also supporting synchronous settings (Priess-Buchheit, 2020; Raes et al., 2020). More related research indicates that during the pandemic a majority of students preferred a synchronous setting. However, asynchronous settings were typically chosen in case of technical difficulties, whereas benefits of synchronous learning are that the students “can interact in real time (Almpanis and Joseph-Richard, 2022).” Emphasizing the property of synchronicity, more related work indicates synchronicity as a “significant role player” (Hepburn and Borthwick, 2021) with a positive overall effect on the (blended) teaching/learning experience, if technically feasible (Katai and Iclanzan, 2023; Belt and Lowenthal, 2023).

#### 3.3.6 Visual representation

Especially in distant settings, the characterization of natural human actors with focus on their visual representation is of great importance. In the worst-case, the representations of all actors significantly differ from the real-world, or are even non-existing (Table 1, “Purely Virtual”). On one hand, such representations are a basic concept for other systems, like multimedia platforms (Herman et al., 2020) or avatars in gaming (Szolin et al., 2023). On the other hand, if the goal is to preserve natural human presence in educational processes with focus on TRA and ACC, this property may be handled with care. If distant and/or asynchronous settings are combined with purely virtual representations of AIS, the room for misinterpretation and misuse may be significantly widened (Figure 3) and must be addressed accordingly (Bardzell et al., 2007). In the worst-case, preventing AIS from mimicking representations of natural human actors may become practically impossible (Figure 4).

#### 3.3.7 Resulting guidelines

**Figure 6:**
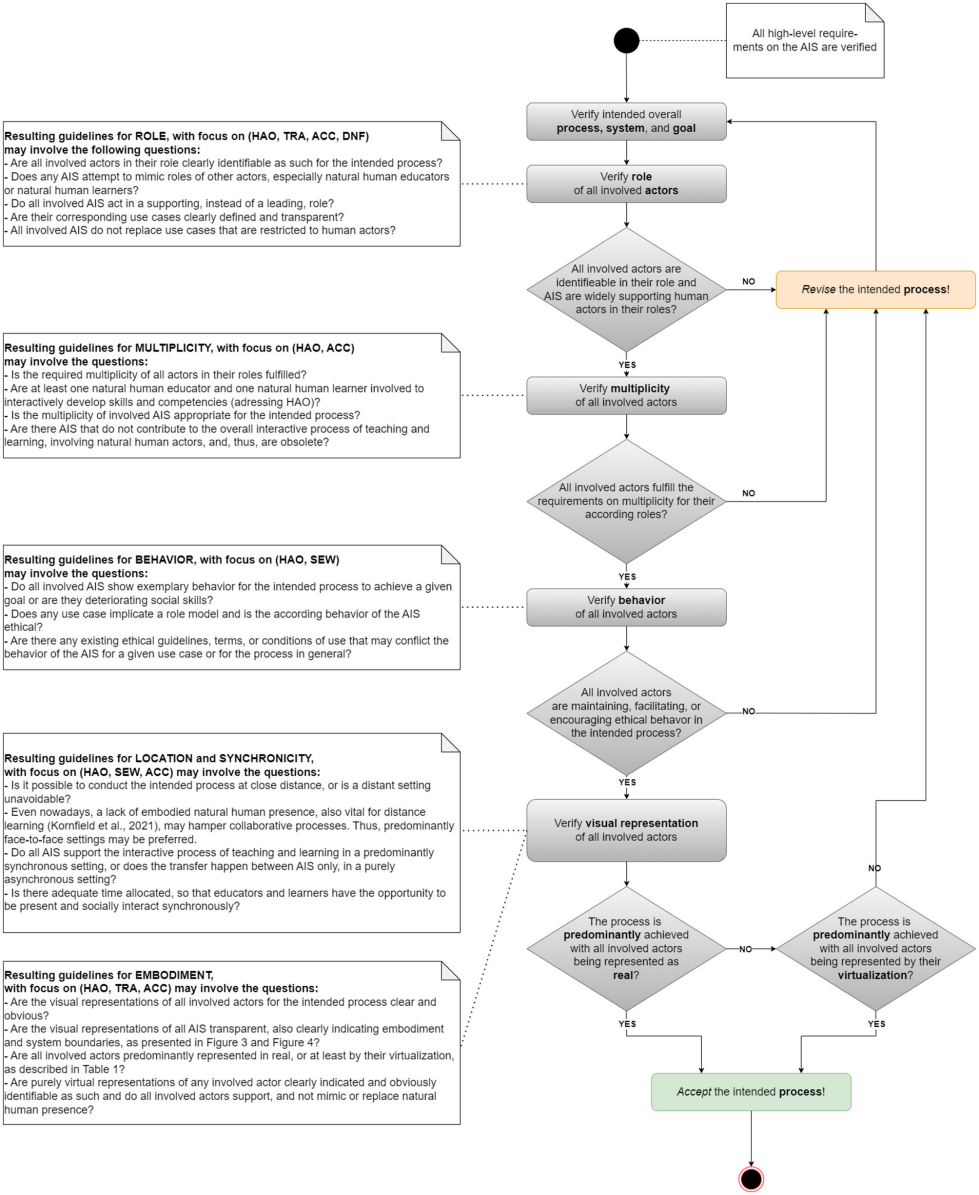
Activity diagram of the conceptual framework, including guidelines to practically verify an ethically justifiable use of AIS for the concrete interactive process of teaching and learning in education.

To better justify the ethical use of AIS in education, the presented low-level framework is substantiated with concrete guidelines, summarized as activity diagram (Figure 6). Whilst concrete questions help to justify potentials for unethical use, the diagram provides practical, and easy to implement yes/no decisions for using AIS as part of an intended process. Thus, clarity regarding an ethical use in education may be drastically increased. The diagram is based on the novel set of properties, derived in Section 3.2 and reflected on in Section 3.3, and relates to typical educational settings modeled in Figure 2.

## 4 Conclusions

This article introduces a conceptual ethical framework with a novel set of low-level properties, particularly highlighting the ethical behavior and the visual representation of the involved actors as core contribution. Whilst a detailed UML model demonstrates the feasibility of according implementation strategies, the goal is to facilitate ethical guidelines, sustainably preserving natural human presence in the use of AIS in education.

### 4.1 Discussion and implications

Reflections on related ethical frameworks revealed the need for a more concrete and detailed modeling of processes, systems and relevant involved actors. General ethical AI frameworks (Rothenberger et al., 2019; Hagendorff, 2020; Fischer, 2022; D́ıaz-Rodŕıguez et al., 2023; Li et al., 2023), or with focus on higher education (Ashok et al., 2022; Schiff, 2022; Ninaus and Sailer, 2022; Nguyen et al., 2023; Chan, 2023; Allen and Kendeou, 2024; Airaj, 2024), are typically high-level and do not investigate on low-level models, properties and according guidelines. Other related studies report on hands on experiences with AIS (Kasneci et al., 2023) and suggest codes of practice (Newton and Newton, 2019), but lack concrete models for implementation. Additionally, state-of-the-art requirements for trustworthy AI, as formulated by the European Commission (EC-Directorate-General-for-Education-Youth-and-Culture, 2022; European-Commission, 2023; HLEG, 2021), lack behavioral and visual low-level properties and according use cases, however which were identified as crucial to facilitate HAO, TRA, SEW, and ACC. Consequently, the presented framework introduces a novel set of low-level properties, particularly stressing the ethical behavior and the visual representation of all involved actors, whereas additional visual indications are suggested to make the system boundaries of AIS more transparent. Although it is hard to cover all ethical implications (Köbis and Mehner, 2021), the framework is enriched with concrete practical guidelines, and a comprehensive workflow. The primary objective is to preserve the presence of natural human actors and foster the interactive process of teaching and learning in the use of AIS. Whilst it is meant as a basis of discussion, it could ultimately help to craft more robust future regulations.

### 4.2 Limitations, challenges, and future research

This article reflects on resulting guidelines that have a strong focus on school education, particularly, pre-schools up to secondary level schools (Motiejunaite-Schulmeister et al., 2022). Whilst the guidelines for multiplicity, location, synchronicity, and visual representation could be more easily implemented for typical school settings, they might become overly restrictive for higher education. Consequently, since universities and a variety of other schools of higher education largely use distant and asynchronous settings (Seo et al., 2021; Leo et al., 2021; Milano et al., 2023; Pregowska et al., 2024), distinguishing between lower and higher education could make the framework significantly more flexible. Other obvious exceptions that would allow for relaxed guidelines might be technical difficulties or lack of appropriate devices (Lassoued et al., 2020). Nevertheless, if technically feasible, guidelines on the visual representation, could in the same way sustainably help to increase HAO, ACC, and TRA for higher education, and thus also increase trust in using AIS (Sathikh et al., 2022; D́ıaz-Rodŕıguez et al., 2023). In this regard, however, preserving natural human presence in distant educational settings is hard to maintain and facilitate, facing future challenges that cannot be entirely addressed in the scope of this article. Ranging from technical limitations or lack of infrastructure (Katai and Iclanzan, 2023; Belt and Lowenthal, 2023) to privacy concerns (Tsai et al., 2020). In those terms, with a variety of other social (Anietor, 2019; Martono et al., 2020; Bintang et al., 2022), cultural, and economic challenges (Wei et al., 2023), it remains unclear up to what scale it will be possible to achieve a sustainable and people focused future of education in the use of AIS. Despite, the presented framework has to face the trade-off between generalization and detail. A more detailed framework indeed increases the effort to frame typical settings in a first step. However, particularly from a sustainable perspective, once relevant properties are identified and a baseline of essential use cases is established, the framework would not need to drastically change amongst various educational settings later on. As a result, future research may address the design of more relaxed models and guidelines for different educational levels. Furthermore, investigating on the application of the ethical framework to an, AIS-supported, teaching and learning system, may help to better clarify practical strengths and weaknesses and, in the same step, could provide a concrete baseline for regulatory conditions of use.

While this article fosters the ethical use of AIS in education, it also represents a dedication to the interactivity and enjoyment inherent in teaching and learning. In that respect it is claimed that preserving natural human presence, in fields with a strong social context, is mandatory. As Floridi (2022) noted, “nobody ever said that doing the right thing was going to be cheap and easy.” In the context of a detailed low-level approach, this article argues that the effort must be justified in the pursuit of a sustainable and human-centric future.

## Footnotes

^1^The symbols of the conceptual visual aid are adapted from “computer” images at Flaticon.com.
